# The Relative Value of the Obstetric Forceps and Version as Methods of Delivery1Read at a meeting of the Obstetrical Society of Philadelphia, April 4, 1907

**Published:** 1907-12

**Authors:** William H. Wells

**Affiliations:** Demonstrator of Clinical Obstetrics in the Jefferson Medical College; Chief Gynecologist to the Mount Sinai Hospital; Assistant Obstetrician to the Jefferson Medical College Hospital


### SELECTED ARTICLES.

### The Relative Value of the Obstetric Forceps and Version as Methods of Delivery.[1]

BY WILLIAM H. WELLS, M. D.,
Demonstrator of Clinical Obstetrics in the Jefferson Medical College; Chief Gynecologist to the Mount Sinai Hospital; Assistant Obstetrician to the Jefferson Medical College Hospital.

(*Therapeutic Gazette*, August 15, 1907)

IN discussing the relative value of the obstetric forceps and version as methods of rapid and safe delivery, we presuppose that the operator has had a fair experience with the use of both

1. Read at a meeting of the Obstetrical Society of Philadelphia, April 4, 1907

methods of delivery, having acquired at least a moderate amount of skill, and where forceps is concerned the instrument used is well made, of good length, and of one of the standard patterns upon which the operator may rely. We mention the latter point because of the following case:

Some four years ago the writer was called one night by a young physician to deliver a primigravidous woman. My young friend in attempting to deliver the patient with a pair of short, light-weight Simpson forceps had broken the anterior blade within the uterine cavity. The forceps did not have an axis traction appliance. He said he thought the piece of blade was in the anterior part of the uterine cavity. When first seen the patient had been in labor about ten hours, was exhausted, and not under an anesthetic. The forceps had been applied because of weak uterine contractions. The head was not engaged, although the pelvic measurements were normal and the head could be brought into the pelvic inlet by pressure. I could not feel the piece of forceps blade by palpation because the bladder was half full of urine. The urine was drawn off, and the patient anesthetised, after which the child's head was gently brought down and rotated far enough to apply the Tarnier forceps. Strong traction was made in a direction downward and especially backward, so as to avoid the piece of forceps blade. The child was born living and well, having suffered no injury whatever; neither was the delivery an especially difficult one. The piece of forceps blade was found on the anterior uterine wall, the broken ends resting downward, as I remember it, against the pubic bone. After withdrawing it I examined the interior of the uterus, and found that no damage whatever had been done. Both mother and child did well.

In the majority of cases we believe that in the mother's interests forceps delivery is safer than version, as far as the operation itself goes. There is less danger of tearing the soft parts of the birth canal, and the slow traction gives the canal time to dilate. In cases, however, in which rapid delivery is necessary in the interests of mother or child, or both, version properly performed will be the better method of delivery.

When weak labor pains delay the expulsion of the child in a patient in whom the fetal vertex is anterior, the os fairly well dilated, and the mother in good condition, stimulants having been tried and having failed, these are the ideal cases for forceps delivery. If the head is engageable above the pelvic floor axis traction should always be used. When the occiput rotates posteriorly to the transverse diameter of the pelvic inlet, it may be rotated slightly anteriorly by the hand, and the forceps, preferably the

Tarnier, may be applied; downward traction in the axis of the pelvic inlet will usually favor anterior rotation of the occiput. Occasionally in this class of cases some sort of loose lock instrument, such as the Simpson, may be used providing the instrument is long enough and has an axis traction appliance. Personally we have had good results in its use. When the occiput has rotated directly backward in pelves of normal size and with no impaction, the forceps should be used in the majority of cases, the Tarnier being here the instrument of choice. Delivery by the forceps where the head is rotated posteriorly takes time, however, and is well adapted for cases in which the child is alive and strong and the mother in good condition. There is a certain proportion of women with slightly flattened pelvis, or instances where mother and child could not stand a long forceps delivery, and in these cases podalic version offers good results providing the soft parts are well dilated.

In flat pelves where the anteroposterior diameter is contracted to such a degree as to retard labor, but not sufficiently to actually prevent the passage of the head, labor may be retarded to such an extent as to cause exhaustion. In such cases the head attempts to enter the pelvis with one of its anteroposterior diameters in relation with the transverse diameter of the inlet, the lateral obliquity of the head being very much increased. In such a position forceps are dangerous; the blades should never be aplied over the forehead and occiput, and the shortened oblique diameters prevent turning the head manually so as to properly apply the forceps. Here podalic version will often save the child's life without materially endangering the mother's. On the other hand, in justominor, generally contracted pelves, the contraction being slight and the head not abnormally large, there is no long transverse diameter in which to turn the child, and in such cases, where the mother cannot deliver herself, the forceps, frequently reënforced by the so-called Walcher's position, will give much better results than version. Labor in both flat and justominor pelves should, however, be most carefully studied: it is so easy to attempt delivery in pelves too small for the child to pass through. External pelvimetry, while some sort of a guide, is not infallible; frequently the external measurements may be normal or nearly so, while the actual diameters of the inlet may be much decreased, due to abnormal thickness of the bones or muscles. In doubtful cases we should not content ourselves with external pelvimetry only, but should measure the internal conjugate by vaginal examination, internal pelvimetry, and by sweeping the finger around the head, which should be also fitted into the pelvic brim. It will be frequently necessary to have the patient under an anesthetic to do this properly.

We repeat that these patients with contracted pelves should be studied with care, and if the operator is doubtful that version or forceps delivery can be safely performed, the patient should be delivered by abdominal incision or craniotomy. The former under proper conditions endangers the patient very little more than does a well-performed forceps delivery in a normal case, and much less than a complicated and lengthy attempt at forceps delivery or version where the pelvis is contracted.

Face presentations, as long as they occur in pelves of normal size, the extension being good and the chin rotating well to the front, usually deliver themselves without help after a slow and tedious first stage. When, however, in primigravidous women whose birth canals are not well dilated or dilate slowly, and uterine contractions are inefficient, the chin may not rotate. In these cases manipulations must be tried to bring the chin to the front of the transverse line, and if these efforts fail podalic version will frequently save the child and shorten the sufferings of the mother by several hours. When the patient is a multiparous woman with well dilated birth canal, slow first stage, the child's head being well extended and the chin anterior, forceps may be used to advantage to cut short a labor which if left to nature might possibly exhaust the patient. The advantage of the Tarnier instrument in this case is that the rotation of the chin may be completed by the rotating action of this instrument. The blades should be accurately fitted to the sides of the child's head. In our own experience we must confess to a preference for podalic version in a majority of these cases.

In shoulder and arm presentations, the head being in one iliac fossa, the breech being in the opposite upper uterine quadrant, much valuable time is lost in trying to push up the head so as to bring it into the inlet sufficiently to apply the forceps; especially is this the case if the membranes have ruptured and the patient has been in labor for some time. Podalic version by the method of Braxton Hicks may here be done with great advantage, the child being more frequently saved.

In cases in which the brow presents, the head being partly extended, birth is impossible. In many of these cases attempts either to flex the head, trying to convert it into a vertex presentation, or to extend and rotate the chin anteriorly, endeavoring to change it into a face presentation, result in failure; thus valuable time is lost. In these cases podalic version skilfully performed offers the child a better chance for life than do prolonged manipulations upon its head, always with the possibility of pressure on the cord and consequent asphyxia.

There can be little doubt as to the value of podalic version in cases of central or partial placenta previa. When, however, the child presents by the vertex and the head is pressing well down on the placenta, it is often better to apply forceps and deliver quickly rather than to run the danger of dislodging the placenta entirely by version. Possibly it may offer the child a rather better chance too. If the head is high up above the pelvic brim, or if the child presents by a part of its body other than the vertex, or there is slow dilatation of the cervix, version should be performed.

Every one who has tried to replace a prolapsed umbilical cord after rupture of the membranes knows how difficult it is to keep the cord in the uterine cavity after replacement, no matter what methods have been used. A large number of children are always lost in these cases. Various methods of delivery have been tried: some operators prefer to place the patient in the knee-chest or Trendelenburg position, replace the cord, then bring down the child's head and extract with forceps. The writer's preference, however, is for podalic version in these cases; we have delivered a larger number of living children by version than by any other method.

Tumors or occlusion of the vagina or uterus, or of the latter extending into the vagina, are contraindications to version, but in a very large number of cases they are to forceps delivery also, so that this class of cases must very largely be treated by abdominal section.

Twice in our experience we have met with cases in which the caput succedaneum was of excessive size. In both instances the mother's pelvis was normal in size or nearly so. The exact cause of such an enlargement of what is usually a small and inoffensive tumor we are unable to state. In one case the tumor projected beyond the bones of the head for a distance of nearly two inches. Application of the forceps resulted in the slipping of the blades. Podalic version was resorted to and gave living children in both cases.

Hemorrhage from a placenta partially separated at its upper margin, the bleeding occurring between the membranes and the uterine wall, constitutes a dangerous complication of pregnancy. Version as a rule in such cases causes the mother to run the additional risk of entirely separating the placenta during the turning of the child. Here the method of preference would be to rupture the membranes, stimulate the uterus to contraction, and deliver the child as soon as the cervix is sufficiently dilated. In a group of cases in which the mother must be delivered with some rapidity because she is the victim of heart, lung, or kidney disease, the method of delivery must depend much on the presentation, the time of pregnancy, and the general condition of the mother and child. As a rule, though, our impression is that less shock will follow a gradual delivery by forceps than by the more rapid one of version and extraction.

CONCLUSIONS.

Where rapid delivery is necessary, the pelvis being of normal size or flattened very slightly in the anteroposterior diameter, the transverse diameter being of normal size or increased, the cervix and vaginal canal dilated and well softened, version is preferable to forceps when mechancial interference is necessary. Among the special indications for version may be classed the following: Brow or face presentations with no rotation of the chin; certain cases of occipitoposterior position; parietal presentations; placenta previa, if the presenting part is high up in the pelvis and the soft parts dilate slowly; certain cases of deficient uterine contractions; prolapse of the cord; transverse positions; excessively large caput succedaneum; some cases of eclampsia.

Forceps delivery gives best results in the largest number of labors delayed by weak uterine contractions where interference is necessary, in the majority of cases of posterior rotation of the occiput. Forceps are also to be used in cases of rachitic, generally contracted, or in justominor pelves, providing the contraction is not great enough to prevent the head and shoulders of the child from passing through the inlet, and in those patients having certain constitutional diseases, when manual delivery is to be performed.

Neither forceps nor version should be attempted in any case of hydrocephalus or abnormally enlarged fetal body unless craniotomy or evisceration has first been performed.

